# Chemical manipulation of hydrogen induced high p-type and n-type conductivity in Ga_2_O_3_

**DOI:** 10.1038/s41598-020-62948-2

**Published:** 2020-04-09

**Authors:** Md Minhazul Islam, Maciej Oskar Liedke, David Winarski, Maik Butterling, Andreas Wagner, Peter Hosemann, Yongqiang Wang, Blas Uberuaga, Farida A. Selim

**Affiliations:** 1 Center for Photochemical Sciences, Bowling Green State University, Bowling Green, Ohio 43403 USA; 2 Department of Physics and Astronomy, Bowling Green State University, Bowling Green, Ohio 43403 USA; 3 Institute of Radiation Physics, Helmholtz-Center Dresden-Rossendorf, Dresden, 01328 Germany; 4 Department of Nuclear Engineering, University of California at Berkeley, Berkeley, CA 94720 USA; 5 Materials Science and Technology Division, Los Alamos National Laboratory, Los Alamos, New Mexico 87545 USA

**Keywords:** Materials science, Physics

## Abstract

Advancement of optoelectronic and high-power devices is tied to the development of wide band gap materials with excellent transport properties. However, bipolar doping (n-type and p-type doping) and realizing high carrier density while maintaining good mobility have been big challenges in wide band gap materials. Here P-type and n-type conductivity was introduced in β-Ga_2_O_3_, an ultra-wide band gap oxide, by controlling hydrogen incorporation in the lattice without further doping. Hydrogen induced a 9-order of magnitude increase of n-type conductivity with donor ionization energy of 20 meV and resistivity of 10^−4^ Ω.cm. The conductivity was switched to p-type with acceptor ionization energy of 42 meV by altering hydrogen incorporation in the lattice. Density functional theory calculations were used to examine hydrogen location in the Ga_2_O_3_ lattice and identified a new donor type as the source of this remarkable n-type conductivity. Positron annihilation spectroscopy measurements confirm this finding and the interpretation of the experimental results. This work illustrates a new approach that allows a tunable and reversible way of modifying the conductivity of semiconductors and it is expected to have profound implications on semiconductor field. At the same time, it demonstrates for the first time p-type and remarkable n-type conductivity in Ga_2_O_3_ which should usher in the development of Ga_2_O_3_ devices and advance optoelectronics and high-power devices.

## Introduction

A wide band gap energy has become a key parameter for the future development of high-power transistors and optoelectronic devices^1^ and wide band gap oxides, such as ZnO, have been shown to exhibit excellent characteristics^2^. However, their deployment in many applications has been hindered due to the lack of conductivity control or the difficulty of realizing high carrier density with good mobility. Bipolar doping (realizing both n-type and p-type) is one of the big challenges in wide band gap materials but it is crucial for most devices^2,3^. Further, substitutional doping of elements, the common method to provide charge carriers, often causes disorder, suppressing carrier mobility and there is always a trade-off between increasing the maximum attainable carrier density and maintaining good mobility in oxides. In this work, we report how to induce p-type and n-type conductivity in an ultra-wide band gap oxide (Ga_2_O_3_) through controlling hydrogen (H) incorporation in the lattice without further substitutional doping and demonstrate a sheet carrier density of 10^16^ cm^−2^ with electron mobility 100 cm^2^ V^−1^ S^−1^ at room temperature leading to 10^−4^ Ω.cm resistivity. Such high electron density and good mobility is remarkable for oxide semiconductors. We identify a new donor concept behind this remarkable conductivity. While recent works reported room temperature electron mobility of 153 cm^2^/(Vs)^4^, 130 cm^2^/(Vs)^5^, and 176 cm^2^/(Vs)^6^ for unintentionally doped β-Ga_2_O_3_ grown by different techniques, the carrier concentrations were significantly low (<10^17^ cm^−3^) in these reports. The same trend is true for Si and Ge doped Ga_2_O_3_^7^ where the carrier concentrations are often low for 100 cm^2^/(Vs) electron mobility. For p-type conductivity we report a hole density of 10^15^ cm^−2^, but with very low hole mobility less than 1 cm^2^ V^−1^ S^−1^, which is expected from the flat valence band of Ga_2_O_3_.

The study was carried out on β-Ga_2_O_3_, as it is emerging as a promising material for high power devices due to its large band gap (~4.5–5 eV) and high breakdown field of 8 MV/cm. It is receiving significant attention in the scientific community as a potential candidate for a wide range of applications^8–13^. β-Ga_2_O_3_ is the most stable polymorph of the Ga_2_O_3_ phases, with a monoclinic crystal structure of space group *C*2*/m*^11^. It behaves as an insulator in its defect free crystalline form. As of today, only one type of conductivity (n-type) has been achieved by doping β-Ga_2_O_3_ with Sn, Ge or Si during growth^14–17^. With respect to p-type conductivity, there has not been any significant success. Only deep acceptors (with activation energy >1 eV) for undoped and doped samples have been reported where acceptor ionization was critically low at room temperature^18,19^.

Hydrogen is known to have a strong influence on the electrical conductivity of semiconductors^20^. In β-Ga_2_O_3_, monoatomic H has a low formation energy and can occupy both interstitial and substitutional sites to act as a shallow donor^21^. The complex crystal structure of β-Ga_2_O_3_ allows for the formation of many configurations where interstitial hydrogen (${{\rm{H}}}_{i}^{+}$) forms a bond with oxygen, creating electronic states which are close in energy. According to J. Varley et.al.^13^, H_i_ acts as a shallow donor, although not stable and substitutional hydrogen, H_O,_ has low formation energy only under oxygen poor condition. Despite these theoretical predictions on the possibility of n-type conductivity due to H-incorporation in various locations, there has not been any report on significant experimental success. In this work, we generate H-donors and H-acceptors in Ga_2_O_3_ by controlling H incorporation on cation vacancy sites, not as H_i_ or H_O_. A cation vacancy is an electrical compensating acceptor in semiconductors including β-Ga_2_O_3_^22^. Although cation vacancies have high formation energy in some oxide semiconductors (e.g. SnO_2_, In_2_O_3_), previous first principle calculations showed that their formation energy is significantly lower in β-Ga_2_O_3_ and hence a high probability of H-decorated V_Ga_ formation can be achieved after incorporating H into the crystal^22,23^.

**Figure 1:**
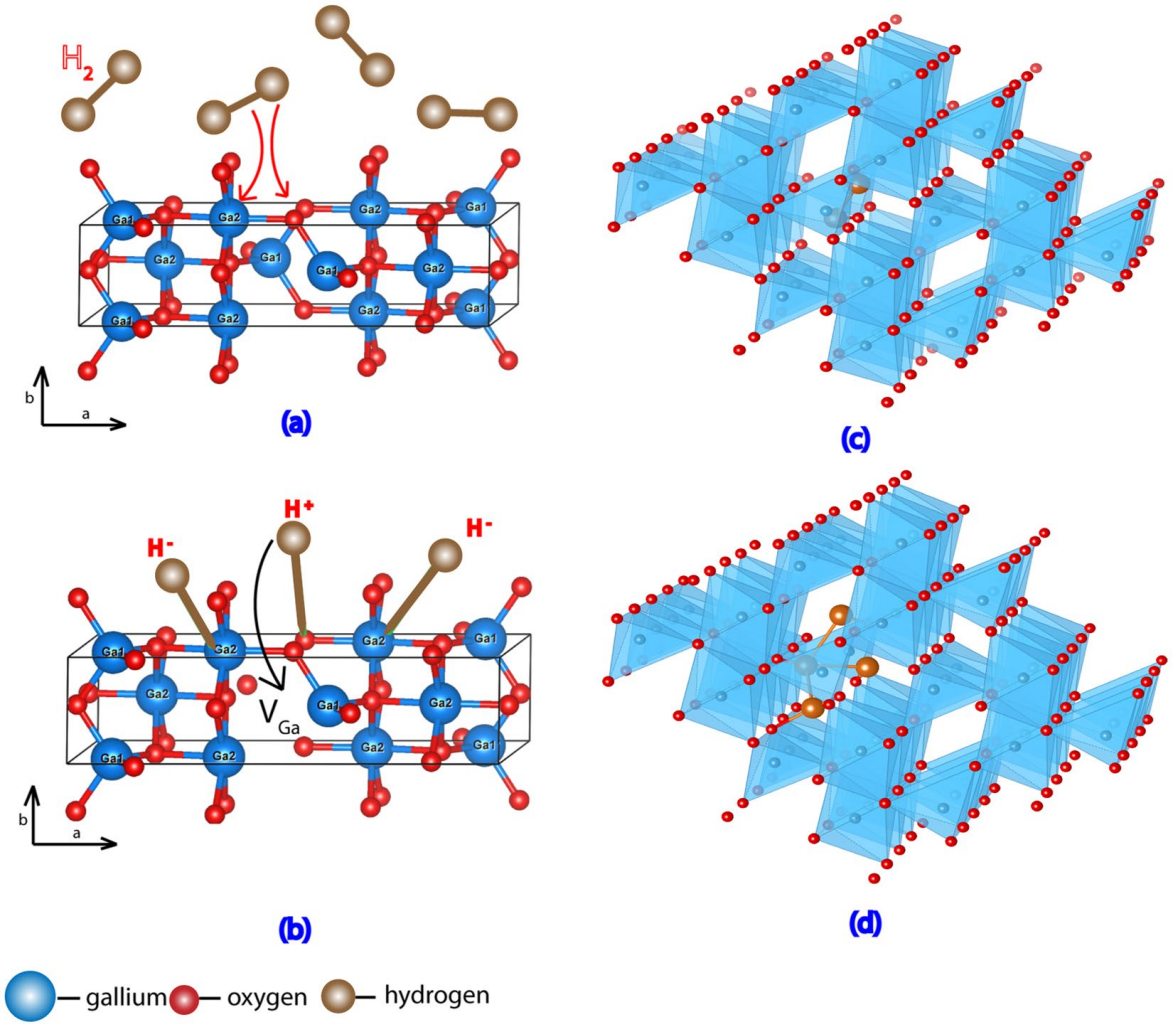
Schematic diagrams showing hydrogen incorporation in β-Ga_2_O_3_ (**a**) hydrogen molecules coming in contact with the surface at elevated temperature and dissociating heterolytically. The electron cloud of H_2_ is attracted toward gallium while the proton is attracted toward oxygen. (**b**) The proton and hydride ion are attached to oxygen and gallium atoms, respectively, on the crystal surface and diffuse through the bulk crystal at high temperatures. The proton is attracted toward the negatively charged gallium vacancy. (**c**) Ga vacancy decorated with two hydrogen as predicted from DFT calculations providing stable acceptor state (**d**) Ga vacancy decorated with four hydrogen as predicted from DFT calculations providing stable donor state.

It is necessary to understand the interaction of H_2_ with the surface of metal-oxide semiconductors to gain insight on the process of H-incorporation into the crystal. H-incorporation into the crystals at high temperature occurs in two steps. At first, H_2_ dissociates and becomes attached to the surface, then diffuses into the bulk crystal. Depending on the nature of the materials, H_2_ can follow either homolytic or heterolytic dissociation pathways. In case of homolytic cleavage, H_2_ molecule dissociates to form two H-atoms that become attached to the oxygen on the crystal surface. On the other hand, H_2_ dissociates to form a proton and a hydride during heterolytic cleavage where the proton and hydride become attached to the oxygen and metal atoms respectively. The redox capacity of metals determines the type of dissociation that is most likely to occur. Density functional theory (DFT) predicts that H_2_ tends to dissociate heterolytically on nonreducible oxide (e.g MgO, γ-Al_2_O_3_) surfaces while following a homolytic pathway on reducible oxide (e.g. CeO_2_) surfaces^24^. β-Ga_2_O_3_ was found to be nonreducible via DFT^25^. Therefore, it is most likely that H_2_ follows heterolytic dissociation as shown in Fig. 1a. The adsorbed proton and hydride diffuse into the bulk crystal at high temperatures. The proton is attracted toward the negatively charged V_Ga_ while the hydride is attracted toward the positively charged or neutral V_O_, as shown in Fig. 1b.

## Results and Discussions

**Figure 2:**
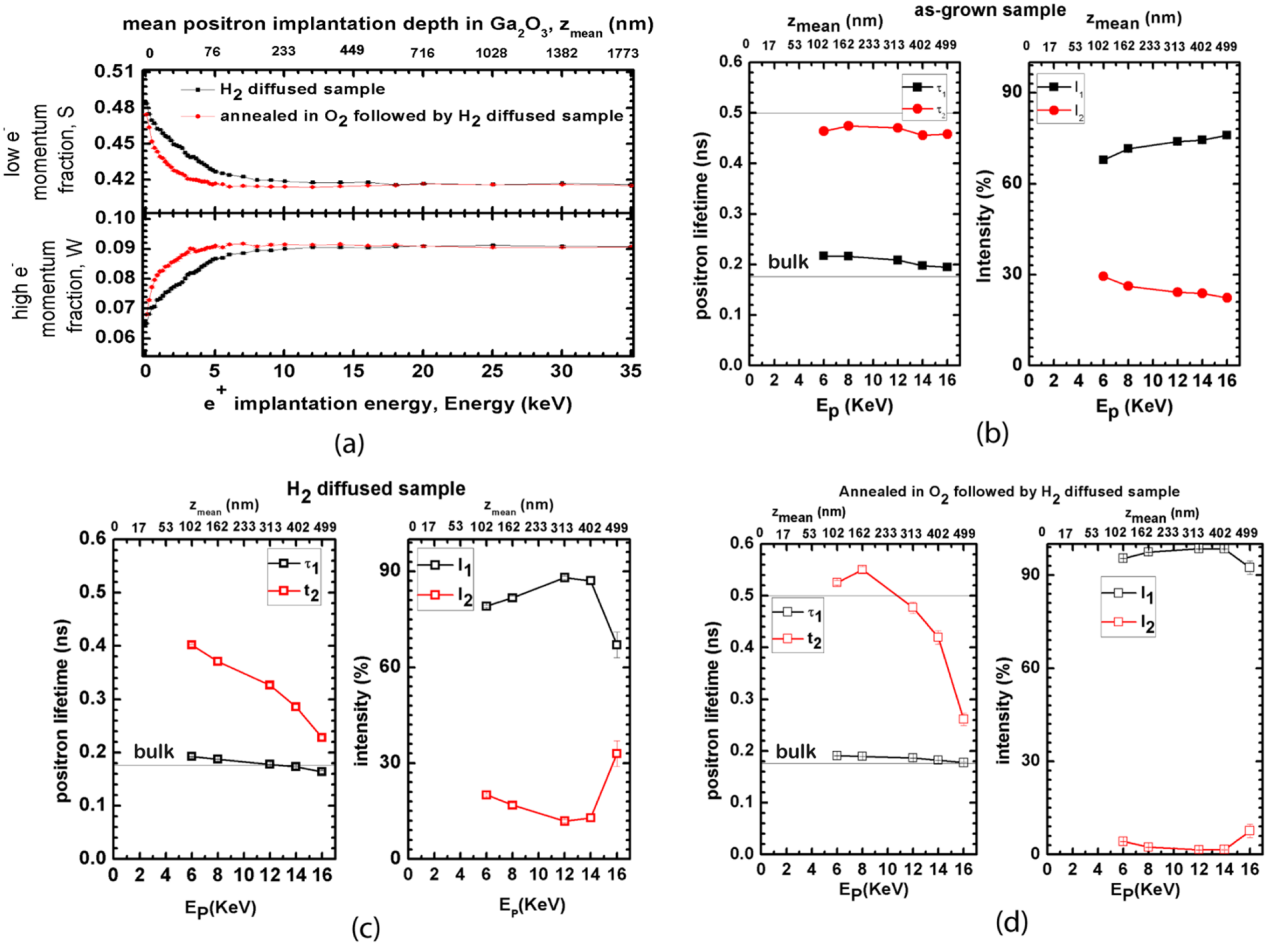
(**a**) Defect parameters S and W measured by Doppler Broadening of Positron Annihilation Spectroscopy (DBPAS) as a function of penetration depth, S and W are defined as the fraction of positrons annihilating with valence and core electrons respectively. The lower x-axis represents the positron energies and the upper x-axis represents the penetration depth. The graph shows that H_2_ diffuses about 500 nm in the crystal. Positron Annihilation Lifetime Spectroscopy (PALS) data of (**b**) as-grown and (c) annealed in H_2_ (950 °C for 2 hours) (**d**) annealed in O_2_ followed by H_2_ (950 °C for 2 hours) samples. E_P_ = Positron implantation energy, Z_mean_ = positron implantation depth, τ = positron lifetime, I = intensity of lifetime component, the graphs (**b, c, and d**) show the two positron lifetime components and their intensities in each sample. τ_1_ is related to the positrons annihilate in the bulk and τ_2_ is related to the positrons annihilate in the defect sites. τ_2_ has significantly decreased after H_2_ diffusion (graph **c**) because of the change of the charge state of the defect center (V_GA_-2H)^−1^. After O_2_ + H_2_ annealing, the contribution of τ_2_ is almost zero because the (V_GA_-4H)^+1^ center has positive charge state and does not trap positrons.

Electrical parameters of the samples measured by Hall measurement system are shown in Table 1. The details of the measurements are given in the method section. Table 1 shows that the as-grown samples were highly resistive, but after H_2_ diffusion they showed an increase in carrier density and p-type conductivity. H_2_ diffusion at 700 °C for 1 hr led to unstable conductivity that decays with time (Table 1a). However, H_2_ diffusion at 950 °C for 2 hrs led to a greater increase in carrier density and stable p-type conductivity over time (Table 1b). Sheet carrier density of 10^15^ cm^−2^ was achieved but hole mobility lower than 1cm^2^/VS was measured which is expected due to the flat band nature of valence band made of predominately O 2p states. Other procedures were carried out to incorporate H_2_ into different sites in the undoped β-Ga_2_O_3_. One sample was annealed in O_2_ flow and another was annealed with Ga in a closed ampoule at 950 °C for 2 hrs. This process should fill up the respective (anion or cation) vacancies. After that, hydrogen was diffused into the crystals at 580 torr in a closed ampoule at 950 °C for 2 hrs. O_2_ –annealing followed by H_2_ diffusion led to high n-type conductivity (stable over time) and remarkable sheet carrier density of about 10^16^ cm^−2^ with electron mobility 100 cm^2^/Vs (Table 1c). The thickness of the conductive layer where H diffuses in is 500 nm as revealed from depth resolved positron measurements in Fig. 2a. This indicates a remarkable conductivity of 10^−4^ Ω.cm. The sample exhibits 9-orders of magnitude increase in conductivity and 10-orders of magnitude increase in carrier density. In contrast, annealing in Ga followed by H_2_ diffusion did not lead to a significant increase in conductivity (Table 1c). Both sole H-diffusion and H-diffusion after O_2_-anneal treatments were carried out on other as-grown undoped Ga_2_O_3_ samples and led to the same results. Samples preserved their p-type or n-type conductivity with no decay or negligible decay after months. The hole sheet number was measured after several months and found to be very 9.4 × 10^14^ cm^−2^ (initial sheet hole number was 1.2 × 10^15^ cm^−2^); after that the carrier concentration seems to be very stable with time with no further decay. The electron sheet number is stable at 10^16^ cm^−2^. As the samples have been processed at very high temperatures, we expect that further processing at high temperature would not be a problem. It is also expected that the samples should sustain high voltage owing to the wide band gap of Ga_2_O_3_. However we have not built a device to test their stability under high voltage, future studies for further device development are necessary and would be highly valuable.

**Table 1:** Transport properties of Ga_2_O_3_ samples measured at room temperature, the thickness of the conductive layer for p-type and n-type is 500 nm measured by depth resolved DBPAS.

sample number	sample	sheet number (cm^−2^)	sheet resistance (ohm/cm^2^)
**(a) H**_**2**_ **diffusion took place in a closed ampoule at 700 °C and 580 torr for one hour**			
1	undoped β-Ga_2_O_3_ single crystal	7.00E + 06	1.940E + 8
2	annealed in H_2_	5.45E + 10 (P- type)	1.480E + 5
3	annealed in H_2_ (after 4 days)	3.44E + 06	7.330E + 8
4	annealed in H_2_ (2nd time)	1.54E + 15 (P-type)	4.060E + 1
5	annealed in H_2_ (2nd time, after 4days)	3.24E + 06	2.360E + 8
**(b) H**_**2**_ **diffusion took place in a closed ampoule at 950 °C and 580 torr for two hours**			
a	undoped β-Ga_2_O_3_ single crystal	5.67E + 06	3.151E + 7
b	annealed in H_2_ (immediately after annealing)	1.20E + 15 (p-type)	1.288E + 1
c	annealed in H_2_ (4 days after annealing)	1.35E + 15 (p-type)	4.126E + 1
**(c) samples annealed in different environments at 950 °C for two hours followed by H**_**2**_ **diffusion at the same temperature and pressure (580 torr)**			
1 or a	as-grown undoped β-Ga2O3 single crystal	5.67E + 06	3.15E + 7
2	annealed in O_2_	2.87E + 06	1.99E + 9
3	annealed in O_2_ followed by annealed in H_2_	6.14E + 16 (n-type)	6.21E + 0
b	annealed in Ga followed by annealed in H_2_	1.55E + 10	2.59E + 5

**Figure 3:**
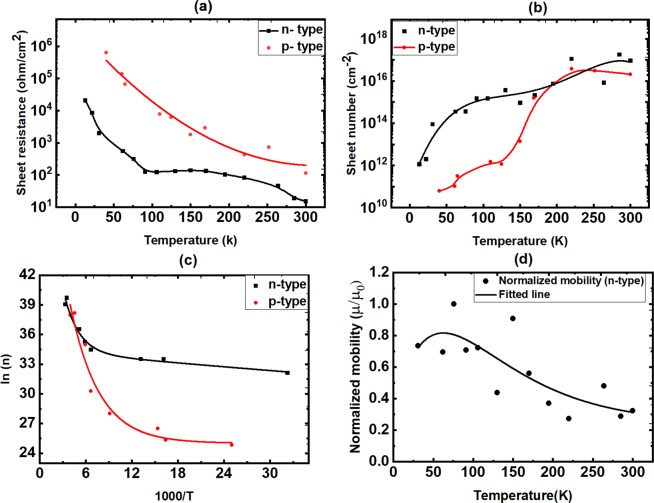
Temperature dependent transport properties of the n-type and p-type H_2_ treated Ga_2_O_3_ samples. (**a**) sheet resistance, (**b**) sheet number, (**c**) sheet number logarithm plotted as a function of 1000/T. (**d**) The dependence of n-type mobility on temperature. The mobility was found to be 100 cm^2^/VS at room temperature; it was normalized to the highest value at low temperature because of the noise in the cryostat system.

Figure 3 shows the temperature dependence of sheet resistance and sheet number of the p-type and n-type Ga_2_O_3_ samples, signifying the ionization of carrier regions followed by extrinsic semiconductor behavior at higher temperatures. Intrinsic semiconductor behavior cannot occur at room temperature as band to band transitions are not possible in Ga_2_O_3_ at this temperature because of the ultra-wide band gap. It is noteworthy to mention that the freeze out regions for the two samples are consistent with the donor/acceptor ionization energy calculated by thermally stimulated luminescence technique presented in later section. It is useful to compare these current measurements by the recent work by Ekaterina *et. al*.^18^ who performed temperature dependent Hall-effect measurements for deep acceptors in unintentionally doped β-Ga_2_O_3_ with ionization energy more than 1 eV, their measurements showed that the activation region is in the range of 300–650 K consistent with deep acceptors. Figure 3 here shows that the activation occurs for donor and acceptor at significantly lower temperatures, which confirms the shallow nature of the induced donor and acceptor states. Figure 3d shows the electron mobility behavior with temperature.

The realization of p-type and n-type conductivity after H_2_ diffusion can be explained as follows. Since the n-type conductivity of the samples was realized after filling up oxygen vacancies and since we know that H-interstitials which act as shallow donors are not stable in Ga_2_O_3_^13^, we attribute the origin of n- and p-type conductivity to hydrogen decorated gallium vacancies, V_Ga-H_. A Ga-vacancy acts as a deep acceptor with −3 charge state (V_Ga_)^3−^. During the diffusion of hydrogen into the crystal, the surface adsorbed proton (Fig. 1a,b) becomes attracted toward the (V_Ga_)^3−^ where it stabilizes the negative charge and, therefore, lowers the acceptor state. This results in H-decorated Ga-vacancy (V_Ga_-2H)^1−^ (as represented in Fig. 1c) and p-type conductivity. At lower temperatures (e.g. 700 °C), protons are less likely to diffuse deep inside the bulk crystal. This results in a decrease in conductivity over time due to the reverse diffusion at room temperature. However, the high p-type conductivity persists over time and becomes stable for the sample exposed to H_2_ at higher temperature and for a longer period of time due to the diffusion of H^+^ deeper into the crystal.

The sample that is exposed to the H_2_ after filling up V_O_ (after annealing in O_2_) showed high n-type conductivity. In this case, more H are diffused into the V_Ga_ due to the absence/reduction of V_O_ leading to the formation of (V_Ga_-4H)^1+^ as represented in Fig. 1d, which acts as a donor. That is, the absence of V_O_ in this case means that the only available traps for H are V_Ga_, which thus become filled to a greater extent. The contribution of n-type conductivity from H_i_ or H_o_ is not prominent as filling up V_Ga_ followed by H-diffusion shows a negligible increase in carrier concentration. Moreover, it confirms that the H-decorated V_Ga_ are primarily responsible for the induced n-conductivity in the samples. It should be also noted that oxygen vacancies are confirmed by now to be deep donors in Ga_2_O_3_ and not source of conductivity^13^.

Density functional theory was used to examine H-incorporation into a Ga-vacancy. The details of the calculation are given in the method section. The results are presented in Table 2. The binding energy of one H^+^ ion to the Ga-vacancy is -4.4 eV. The DFT calculations reveal that, as N (the number of H ions) increases, at least up to N = 4, the reaction remains exothermic, though the strength of the binding, per H atom, decreases. The energy gained by adding the 4^th^ H^+^ ion is only −0.8 eV, much less than the −4.4 eV gained by adding the 1^st^ H^+^ ion. If the trend persisted, this suggests that no more than 4 H^+^ ions can be favorably accommodated into V_Ga_. Thus, these calculations indicate that a single V_Ga_ can accommodate up to 4 H^+^ ions, changing the net charge of the complex from 3- (when N = 0) to 1+ (when N = 4), and confirm that (V_Ga_-4H)^1+^ (Fig. 1d) is more favorable than H_i_^+^. These calculations verified our interpretation of the electrical transport measurements that (V_Ga_-4H)^1+^ is the dominant donor in the treated highly conductive n-type sample. This cation vacancy filled with the relevant numbers of H^+^ represents a new type of donor that does not create disorder in the lattice suppressing electron mobility as in the case of standard dopants on substitutional or interstitial sites.

**Table 2:** Binding energy of H^+^ ions to a Ga vacancy.

N	Net charge of the H-V_Ga_ complex	Binding energy (eV)	Binding energy per H (eV)	Binding energy of extra H (eV)
1	−2	−4.4	−4.4	−4.4
2	−1	−7.5	−3.7	−3.1
3	0	−9.4	−3.1	−1.9
4	+1	−10.2	−2.6	−0.8

**Figure 4:**
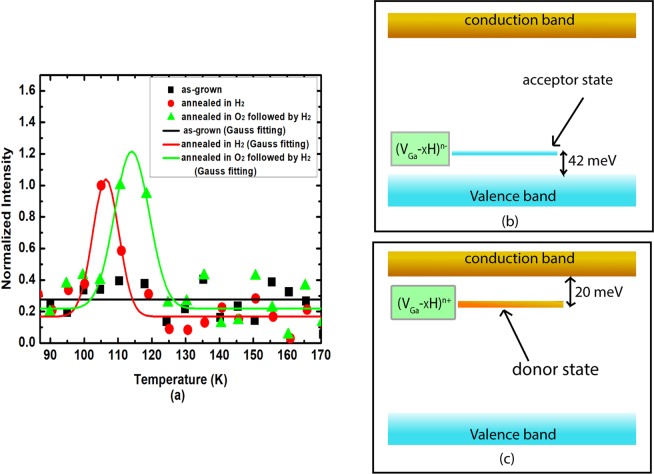
Thermally stimulated luminescence emission (**a**) of the samples annealed at 950 °C for two hours in different environments and the as-grown sample. Data points for annealed samples were normalized from 0 to 1. Data points for as-grown sample were normalized from 0 to 0.5 to minimize noise (no glow peak). Peaks were fitted with a Gaussian function. The two peaks appeared at low temperature after H_2_ diffusion, and after O- anneal followed by H-diffusion are associated with the induced shallow acceptor and shallow donor in the samples respectively and they were used for calculating the ionization energies. The flat band diagrams showing donor and acceptor states of the samples after direct hydrogen diffusion (**b**) and hydrogen diffusion after filling up oxygen vacancies (**c**).

Low temperature thermally stimulated luminescence spectroscopy was performed on the samples to reveal the shallow donor/acceptor levels. At low temperatures, shallow donor/acceptor level can be provided with carriers by optical injection that get trapped at respective levels. These carriers can be released by thermal excitation that reveals valuable information about the shallow donor/acceptor levels. The details of the experiments can be found in the method section and in the text and Supplementary Figure 1 in the Supplementary Information file. Figure 4a displays the TSL emission for as-grown, p-type and n-type H_2_ treated Ga_2_O_3_. The as-grown sample shows no peak corresponding to shallow levels. Each of the other two samples shows a peak at low temperature indicating the formation of shallow level. The peak formed at 107 K in the p-type H_2_-anneal sample (red curve in Fig. 4a) is associated with the formation of shallow acceptors with ionization energy of 42 meV, calculated using the simplified model of TL developed by Randal and Williams^26–28^. The ionization energy of the donor, emerging after O_2_-annealing followed by H_2_-diffusion (green curve in Fig. 4a), was also calculated by the initial rise method from the peak at 111 K and found to be 20 meV. Figure 4b,c shows the corresponding flat band diagram and corresponding donor and acceptor state. The details of the calculation of donor/acceptor ionization energy is provided in the text and Supplementary Figure 2 in the Supplementary  Information.

To further understand the effect of H-incorporation and confirm our interpretation of the origin of conductivity, we carried out Positron Annihilation Spectroscopy (PAS) measurements. The details of the experiments and data analysis are given in the method section. Positron Annihilation Spectroscopy is a powerful technique to investigate cation vacancy type defects^29^. It has also been established as an effective tool to probe the incorporation of hydrogen in cation vacancies as partial or complete passivation of vacancies by hydrogen strongly impact positron trapping^30–32^. We have performed Positron Annihilation Lifetime Spectroscopy (PALS) and Doppler Broadening of Positron Annihilation Spectroscopy (DBPAS), two varieties of Positron Annihilation Spectroscopy that gives valuable information about the electronic environments in cation vacancy sites. Figure 2a presents the defect parameters S and W (defined in Fig. 2) as a function of depth for the two treated samples. The large values of S at the very beginning of the two curves are common in all DBPAS measurements, indicating the formation of positronium at the surface. The graph shows a large difference between the two samples only in the first 500 nm (where H diffuses in) with lower S values and higher W values for the sample annealed in O_2_ followed by H_2_, which exhibits high n-type conductivity. The decrease in S-parameter is an indication for the suppression of positron trapping at cation or neutral vacancies. Thus, these measurements confirm the decrease of negatively charged and neutral vacancies in the O_2_-annelead followed by H_2_-diffusion sample. This must be due to filling of Ga-vacancies with more than three H-ions leading to a positive charge state and the formation of a shallow donor as indicated by the immense increase in n-type conductivity. This (H-V_Ga_)^1+^ complex has a positive charge state and cannot trap positrons, leading to the substantial decrease in S-parameter. On the other hand, sole H_2_-diffusion leads to partial filling of V_Ga_ with hydrogen maintaining a negative charge state and leading to shallow acceptors, which imparts p-type conductivity. This (H-V_Ga_)^1−^ complex is still an active positron trap which leads to a higher S-value.

Depth resolved measurements of PALS revealed two major positron lifetime components for each sample (example of PALS spectra measured for the p-type and n-type samples is given in Supplementary Figure 3 in the Supplementary Information file). Figure 2b–d show the lifetime components and their intensity as a function of depth for the as-grown sample, and the H_2_ diffused, and O_2_-annealed followed by H_2_-diffused samples. A distinctive difference can be seen in the intensity and magnitude of the positron lifetime components among the three samples. The large second lifetime component τ_2_ indicates the presence of V_Ga_-related defects with negative charge states. For as-grown Ga_2_O_3_, τ_2_ is about 470 ps with about 25 to 30% intensity across the sample depth (Fig. 2b). After H_2_-anneal, τ_2_ was reduced to ~320 ps indicating partial filling of V_Ga_ related defects with hydrogen while its intensity was reduced to about 13% (Fig. 2c) due to the decrease of positron trapping at these vacancies as result of less negativity. After annealing in O_2_ followed by H_2_-diffusion, almost all positrons annihilate with lifetimes close to the bulk lifetime (Fig. 2d)^33^. The intensity of τ_2_ was reduced to about less than 1% indicating almost complete absence of positron trapping at defects providing strong evidence for filling up V_Ga_ related defects with H_2_ transforming them into donors with a positive charge state, which cannot trap positrons. Thus, DBPAS and PALS measurements explicitly confirm our interpretation for the origin of n-type and p-type conductivity.

Precise doping and carrier control is important to realize β-Ga_2_O_3_ based bipolar devices^34,35^. In this work, we found that the best way to control the number of H in V_Ga_ is to remove V_O_ and keep H_2_ pressure the same. By doing this, we can increase the number of H incorporating in the V_Ga_. Here, the number of H resides in the V_Ga_ is governed by the thermodynamic stability of the complex and the availability of hydrogen atoms. However, the number of H incorporated in the V_Ga_ can also be controlled by adjusting H_2_ pressure. A detail study of the effect of hydrogen pressure on the type and concentration of carriers would be of great interest to further develop different processes for H incorporation.

## Conclusions

In summary, by controlling H-incorporation in the lattice, we have demonstrated the development of stable p-type and n-type Ga_2_O_3_, which is expected to significantly advance optoelectronics and high-power devices. In the meantime, we illustrated a potential simple method for tuning and switching the conductivity of semiconductors between p-type and n-type with the realization of remarkable high carrier density and good mobility in wide band gap oxides, which is a significant challenge by common substitutional doping methods. A concept for new donor type as cation vacancy filled with the relevant numbers of H^+^ was introduced and found to be behind the remarkable n-type conductivity. This new donor type does not create disorder in the lattice, which often suppresses carrier mobility in the case of standard doping.

## Methods

### Hydrogen incorporation process

High quality β-Ga_2_O_3_ samples grown by Edge- defined Film-fed Growth (EFG) method were obtained from Tamura Inc., Japan. A number of undoped highly resistive samples (5 mm × 5 mm × 0.5 mm) were selected and placed in a quartz ampoule with one open end that was connected to a vacuum pump to pump the air out and evacuate the ampule. After that, the tube was filled with H_2_ gas at 580 torr pressure. After filling the tube with hydrogen, the open end was properly sealed. The ampoule was placed in an oven where temperature can be precisely controlled. The temperature was increased in two steps up to the desired value and H_2_ was allowed to diffuse into the crystal for 1 or 2 hours. A few other samples of same dimensions were first annealed in oxygen flow at 950 °C and then hydrogen following the same procedure, while others were annealed first with gallium, then hydrogen following the same procedure.

### Hall-effect measurements

Van der Pauw Hall-effect measurements were performed to determine the electrical transport properties of the samples. The measurements were carried out from 30 K to room temperature (298 K) and at constant magnetic field of 9300 G. Four indium contacts were made in a square arrangement on the surface of each sample and carefully adjusted to keep the contacts as small as possible. Current-voltage linearity was checked every time to make sure that the contacts were good and resistivity does not vary more than 10% between different contact points. Temperature dependent measurements of the carrier concentration were carried out from 30 K or below to room temperature using a closed cycle cryostat.

### Computational analysis

Density functional theory, as implemented in the Vienna ab-initio Simulation Package (VASP)^36,37^, was used to examine H-incorporation into a Ga-vacancy. These calculations were performed on a 1 × 4 × 2 supercell of β-Ga_2_O_3_, containing a total of 160 atoms in the defect-free structure. A Г-centered 2 × 2 × 2 Monkhorst-Pack k-point mesh^38^ was used to sample the Brillouin zone. The energy cutoff for the planewaves was 400 eV. Pseudopotentials based on the projector augmented wave method^39^ and the Perdew, Burke, and Ernzerhof (PBE)^40^ generalized gradient approximation (GGA) exchange-correlation functional (which should be good enough for our purpose here) were used. Calculations were continued until the maximum component of the force on any atom was less than 0.02 eV/angstrom, with one exception (the charged Ga-vacancy), where such a tight convergence was not possible. In this case, the maximum force was 0.024 eV/angstrom. Both monopole corrections (using a calculated dielectric constant of 4.16, which is a bit higher but similar to previously reported values)^41^ and an alignment correction were applied to the energies. Instead of averaging the potential to perform the alignment correction, we simply shifted the density of states such that the deepest state in the material aligned across different structures, which has been shown to give similar corrections^42^. In any case, the magnitude of this correction was no greater than 0.1 eV.

A V_Ga_ was created by removing a tetrahedrally-coordinated Ga ion from the cell, as this vacancy structure has been identified as being more favorable^43^. A net charge of −3 was imposed on the structure. H^+^ ions with a charge of +1 were inserted into the resulting vacancy structure (leaving the total number of electrons in the system constant but reducing the net charge of the cell). The resulting binding energy for each configuration was computed via the following relationship:

1 $${E}_{b}=E({[{V}_{{\rm{Ga}}}{\rm{NH}}]}^{(-3+N)})+E({\rm{Bulk}}\,{{\rm{Ga}}}_{2}{{\rm{O}}}_{3})E({{V}_{{\rm{Ga}}}}^{3-})-NE({{\rm{H}}}^{+})$$

where $E({[{V}_{{\rm{Ga}}}{\rm{NH}}]}^{(-3+N)})$ is the energy of the system with the Ga vacancy filled with *N* H^+^ ions, $E({\rm{Bulk}}\,{{\rm{Ga}}}_{2}{O}_{3})$ is the energy of defect-free β-Ga_2_O_3_, $E({{V}_{{\rm{Ga}}}}^{3-})$ is the energy of the isolated Ga vacancy in a 3- charge state, and $E({H}^{+})$ is the energy of an isolated 1+ H interstitial. With this definition, a negative energy indicates an exothermic or favorable reaction. We did not perform a systematic search for the lowest energy H interstitial position but performed multiple minimizations where the H was randomly displaced to find a reasonable structure. The structure found here, in which the H^+^ ion is bonded to one of the three-fold coordinated oxygen ions, is similar to that described by Varley *et al*.^13^.

### Thermal stimulated luminescence spectroscopy (TSL)

Thermal stimulated luminescence (TSL) spectroscopy^26–28,44–46^ was performed on the samples to calculate the donor and acceptor ionization energies^45^. The measurements were performed using an in-house built spectrometer^26,47^, from −190 °C to 25 °C. The samples were first placed in a dark compartment and irradiated with UV light at −190 °C for 30 min. After irradiation, the temperature of the samples was set to increase at constant rate (60 °C/min) and the emission spectra were recorded from 200 to 800 nm at every 5 seconds. The glow curves which represent the emission intensity as a function of temperature were constructed from the integration of emission over wavelengths at each temperature.

### Positron annihilation spectroscopy

We carried out positron annihilation spectroscopy (PAS), which is a well-established technique to detect and characterize cation vacancies in semiconductors and oxides^48,49^. Both Doppler Broadening of Positron Annihilation Spectroscopy (DBPAS) and Positron Annihilation Lifetime Spectroscopy (PALS) were employed. DBPAS measurements were carried out using a mono-energetic variable energy positron beam at Helmholtz-Zentrum Dresden-Rossendorf (HZDR) facility in Dresden, Germany^50^. Positrons are emitted from an intense ^22^Na source and a tungsten moderator and accelerated to discrete energy values E_p_ in the range of E_p_ = 0.05–35 keV. Such positron implantation energy, E_p_ allows penetrating up to about 1.8 µm in Ga_2_O_3_. Doppler broadened spectra representing positron annihilation distribution for each E_p_ were acquired using a single high-purity germanium detector with energy resolution of 1.09 ± 0.01 keV at 511 keV and the S and W parameters (defined in the caption of Fig. 2) were calculated from the peak. PALS has been established as the most effective method to probe cation vacancy related defects, distinguishing between their types and providing information about their concentrations^49^. PALS was performed at the Mono-energetic Positron Spectroscopy (MePS) pulsed beam, which is the end station of the radiation source ELBE (Electron Linac for beams with high Brilliance and low Emittance) at HZDR facility in Dresden Germany^50^. The lifetime spectrum was measured at each positron energy E_p_ up to 16 keV with a time resolution of 205 ps. All lifetime spectra contained at least 5 × 10^6^ counts and were analyzed as a sum of time-dependent exponential decays, *N(t*) *= Σ*_*i*_ *I*_*i*_*/τ*_*i*_*·exp(−t/τ*_*i*_*)* convoluted with the Gaussian’s functions describing the spectrometer timing resolution, using the PALSfit fitting software^51^. Depth-resolved measurements of PALS revealed two major positron lifetime components for each sample.

## Supplementary information

Supplement material.
