# Supplementary material for: Chemical manipulation of hydrogen induced high p-type and n-type conductivity in Ga2O3
Source: Sci Rep. 2020 Apr 9;10:6134. doi: 10.1038/s41598-020-62948-2 (PMC7145873; doi:10.1038/s41598-020-62948-2)
Supplement: Supplementary file 1 — Supplement material. [file 41598_2020_62948_MOESM1_ESM.pdf]

Supplementary Information

Chemical manipulation of hydrogen induced high p-type and n-type conductivity in Ga_2O_3

Md Minhazul Islam[1,2], Maciej Oskar Liedke[3], David Winarski[1,2], Maik Butterling[3], Andreas Wagner[3], Peter Hosemann[4], Yongqiang Wang[5], Blas Uberuaga[5] and Farida A. Selim[1,2 *]

[1]Center for Photochemical Sciences, Bowling Green State University, Bowling Green, Ohio 43403, USA.

[2]Department of physics and astronomy, Bowling Green State University, Bowling Green, Ohio 43403, USA.

[3]Institute of Radiation Physics, Helmholtz-Center Dresden-Rossendorf, Dresden 01328, Germany.

[4]Department of Nuclear Engineering, University of California at Berkeley, Berkeley, CA 94720, USA.

[5]Materials Science and Technology Division, Los Alamos National Laboratory, Los Alamos, New Mexico 87545, USA.

*Corresponding author: faselim@bgsu.edu

Supplementary Discussion 1:

Calculations of Donor/Acceptor ionization energy by Thermoluminescence Spectroscopy

This section provides details about the calculations of donor/acceptor ionization energy by Thermoluminescence Spectroscopy.

Thermoluminescence (TL) is the emission of light from materials upon thermal stimulation after irradiating the sample by ionizing radiation at low temperatures. It is a powerful technique to calculate the energy levels of defects that trap charge carriers (e.g. electrons/hole) at low temperature. The phenomena can be explained by energy band theory of solids.[1] At lower temperatures, most of the charge carriers (e.g. electrons/holes) reside in the valence band in an ideal semiconductor. Electrons can be excited to the conduction band (holes to the valence band) upon excitation. Wide band gap materials often have structural defects that can trap charge carriers. Donor/acceptor states can also be thought of as defects that trap charge carriers at low temperatures. Thermal stimulation can release the electrons/holes from these traps where they transfer their energy to luminescence centers. A schematic diagram of TL process is given in supplementary figure 1 for donor and acceptor cases.

Donor/acceptor ionization energy was calculated by initial rise method.[1] Randall and Wilkins simplified the thermoluminescence model by assuming negligible re-trapping, linear heating rate and formulated the well-known Randall–Wilkins first order expression for TL intensity[1]

$$I(T) = n_0 \frac{s}{\beta} exp\left\{-\frac{E_D}{kT}\right\} \times exp\{-\frac{s}{\beta}\int_{T_0}^{T} exp\left\{-\frac{E_D}{kT\prime}\right\} dT' \quad (1)$$

Here, s is the frequency factor and is considered as a constant in the simplified model, T is the absolute temperature, k is Boltzman constant and E_D is the donor/acceptor ionization energy, n_0 is

the total number of trapped electrons/holes at time t=0, β is the constant heating rate. The symmetric shape of the peaks for our samples indicates second or higher order kinetics where significant re-trapping of charge carrier occurs after de-trapping from the traps. A similar equation was derived for the second order kinetics where significant re-trapping occurs.[2]

$$I(T) = \frac{n_0^2 s}{N\beta} exp\left\{-\frac{E_D}{kT}\right\} \times [1 + \frac{n_0 s}{N\beta}\int_{T_0}^{T} exp\left\{-\frac{E_D}{kT'}\right\} dT']^{-2} \quad (2)$$

Initially, intensity of glow peak is dominated by the first exponential half of these equations [equations (1), (2)] and the last half can be negligible. As a result, if ln(I) is plotted as a function of 1/T for the initial points of the glow peak, a straight line is obtained with the slope from which donor/acceptor ionization energy, E_D, can be calculated. Linear fittings of ln(I) vs 1/T for n-type (Fig. 2a) and p-type (Fig. 2b)) samples are shown in Supplementary figure 2. Donor ionization energy of (a) β-Ga_2O_3 sample annealed in oxygen followed by hydrogen diffusion and acceptor ionization energy of (b) hydrogen diffused β-Ga_2O_3 sample were found to be 20 meV and 42 meV respectively.

REFERENCES

[1]A. J. J. Bos, 'High sensitivity thermoluminescence dosimetry', *Nucl. Instr. Meth. Phys. B* 184, 3-28 (2001).

[2]C. Greskovich and S. Duclos, 'Ceramic scintillators' *Annu. Rev. Mater. Sci.* 27, 69 (1997).

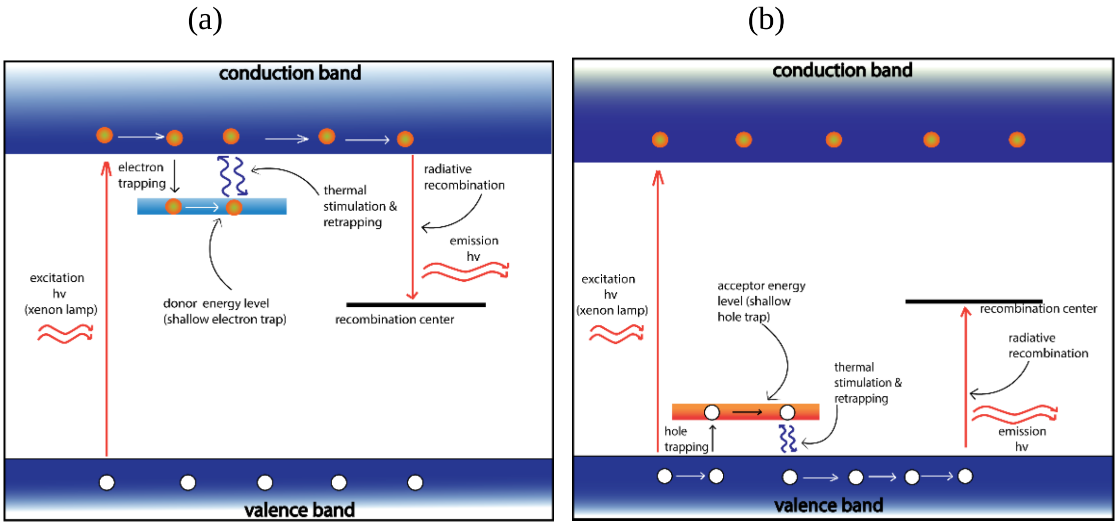

Supplementary figure 1: Schematic diagram of Thermoluminescence process for (a) donor and (b) acceptor case

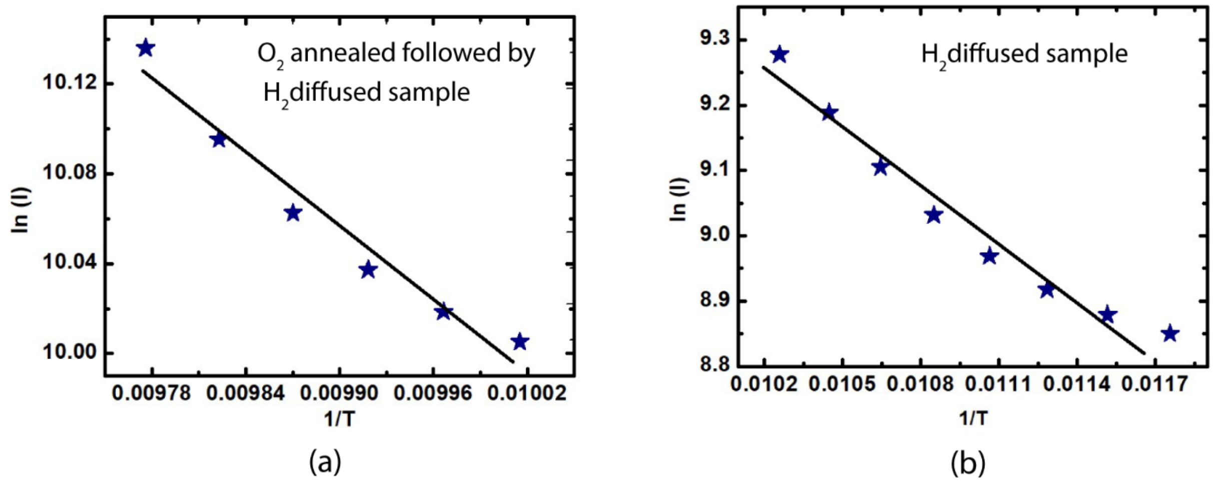

Supplementary figure 2: Calculation of ionization energy by Initial Rise method. Linear fitting of ln (I) vs 1/T of (a) β-Ga_2O_3 sample annealed in oxygen followed by hydrogen diffusion (b) hydrogen diffused β-Ga_2O_3 sample.

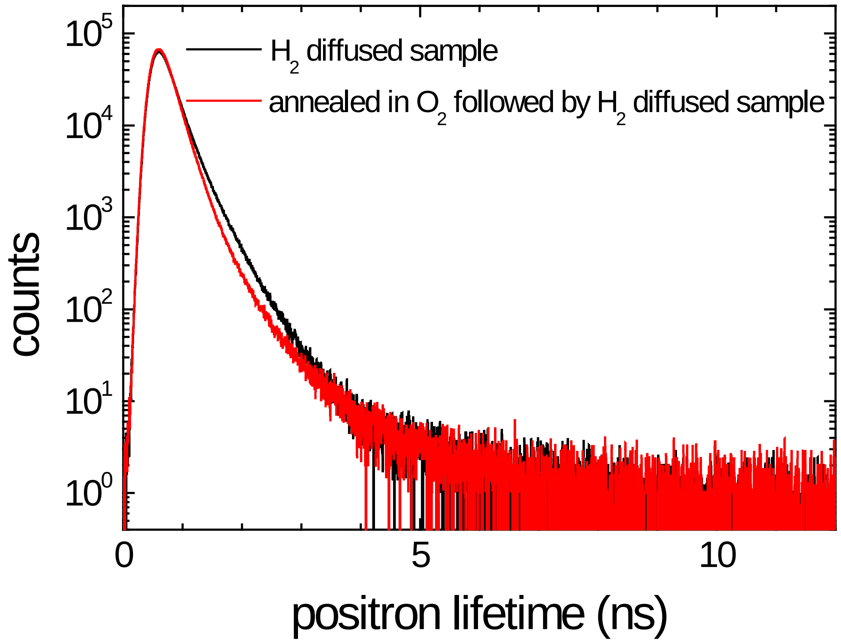

Supplementary figure 3: Positron lifetime spectra at E_p=6 keV for the H_2 diffused sample and the sample annealed in O_2 followed by H_2 diffusion
